# Comparative genome analysis of rice-pathogenic *Burkholderia* provides insight into capacity to adapt to different environments and hosts

**DOI:** 10.1186/s12864-015-1558-5

**Published:** 2015-05-06

**Authors:** Young-Su Seo, Jae Yun Lim, Jungwook Park, Sunyoung Kim, Hyun-Hee Lee, Hoon Cheong, Sang-Mok Kim, Jae Sun Moon, Ingyu Hwang

**Affiliations:** Department of Microbiology, Pusan National University, Busan, 609-735 Republic of Korea; Department of Agricultural Biotechnology, Seoul National University, Seoul, 151-921 Republic of Korea; Plant Quarantine Technology Center, Animal and Plant Quarantine Agency, Suwon, 443-400 Republic of Korea; Yeongnam Regional Office, Animal and Plant Quarantine Agency, Busan, 600-016 Republic of Korea; Korea Research Institute of Bioscience and Biotechnology, Daejeon, 305-633 Republic of Korea

**Keywords:** *Burkholderia gladioli*, *B. glumae*, *B. plantarii*, Comparative genomics, Pathogen, Rice

## Abstract

**Background:**

In addition to human and animal diseases, bacteria of the genus *Burkholderia* can cause plant diseases. The representative species of rice-pathogenic *Burkholderia* are *Burkholderia glumae, B. gladioli,* and *B. plantarii*, which primarily cause grain rot, sheath rot, and seedling blight, respectively, resulting in severe reductions in rice production. Though *Burkholderia* rice pathogens cause problems in rice-growing countries, comprehensive studies of these rice-pathogenic species aiming to control *Burkholderia*-mediated diseases are only in the early stages.

**Results:**

We first sequenced the complete genome of *B. plantarii* ATCC 43733^T^. Second, we conducted comparative analysis of the newly sequenced *B. plantarii* ATCC 43733^T^ genome with eleven complete or draft genomes of *B. glumae* and *B. gladioli* strains. Furthermore, we compared the genome of three rice *Burkholderia* pathogens with those of other *Burkholderia* species such as those found in environmental habitats and those known as animal/human pathogens. These *B. glumae*, *B. gladioli*, and *B. plantarii* strains have unique genes involved in toxoflavin or tropolone toxin production and the clustered regularly interspaced short palindromic repeats (CRISPR)-mediated bacterial immune system. Although the genome of *B. plantarii* ATCC 43733^T^ has many common features with those of *B. glumae* and *B. gladioli*, this *B. plantarii* strain has several unique features, including quorum sensing and CRISPR/CRISPR-associated protein (Cas) systems.

**Conclusions:**

The complete genome sequence of *B. plantarii* ATCC 43733^T^ and publicly available genomes of *B. glumae* BGR1 and *B. gladioli* BSR3 enabled comprehensive comparative genome analyses among three rice-pathogenic *Burkholderia* species responsible for tissue rotting and seedling blight. Our results suggest that *B. glumae* has evolved rapidly, or has undergone rapid genome rearrangements or deletions, in response to the hosts. It also, clarifies the unique features of rice pathogenic Burkholderia species relative to other animal and human Burkholderia species.

**Electronic supplementary material:**

The online version of this article (doi:10.1186/s12864-015-1558-5) contains supplementary material, which is available to authorized users.

## Background

The genus *Burkholderia* contains over 40 species, which occupy diverse niches and are found in a range of environmental habitats, including soil and water, and even in the hospital setting. *Burkholderia* organisms act as pathogens, endophytes, and symbionts [1,2]. Although many members of the genus are plant pathogens and soil bacteria, the most comprehensive characterizations of *Burkholderia* species have been conducted on those organisms that are opportunistic human pathogens [3]. One of two major human-infectious *Burkholderia* groups comprises *B. mallei* and *B. pseudomallei*, the causative agents of glanders and melioidosis, respectively. The other major group of *Burkholderia* human pathogens is *B. cepacia* complex bacteria, which are associated with severe infections in individuals with cystic fibrosis. Recently, increasing numbers of *Burkholderia* species have been reported as plant-associated bacteria.

*Burkholderia* species can be free-living in the plant rhizosphere, or can reside within plants as endophytes or symbionts. Some *Burkholderia* strains are known to aid plants by enhancing disease resistance, improving nitrogen fixation, and enabling adaption to environmental stresses [4-6]. However, there is little information regarding plant-pathogenic (phytopathogenic) *Burkholderia* species, with the exception of *B. glumae. B. glumae* causes grain rot in rice, and is used as a model system of quorum sensing (QS) mechanisms in gram-negative phytopathogenic bacteria [7-10]. Two other important phytopathogenic *Burkholderia* species, *B. gladioli* and *B. plantarii*, are pathogenic to rice and are primarily responsible for sheath rot and seedling blight, respectively [11,12]. Under the right environmental conditions, these three pathogenic *Burkholderia* species can cause severe damage to rice crops in various developmental stages.

In addition to occupying remarkably diverse niches, the genomes of *Burkholderia* species range greatly in size, from ~3.75 to 11.29 Mbp. Among *Burkholderia* organisms, *B. rhizoxinica* (a bacterial endosymbiont of the fungus *Rhizopus microsporus*) harbors the smallest genome (~3.75 Mbp), and the soil bacterium *B. terrae* has the largest genome (~11.5 Mbp). The first *Burkholderia* rice pathogen to have its complete genome sequenced was *B. glumae* BGR1 [13], and the genome of *B. gladioli* BSR3 was subsequently sequenced [14]. The genomes of *B. glumae* and *B. gladioli* both consist of two chromosomes and four plasmids, with genome sizes of 7.09 Mbp and 9.05 Mbp, respectively. Recently, comparative genome analysis of two *B. glumae* strains from different geographic regions showed high degree of genomic variation [15] and genetic differences between *B. glumae* and *B. gladioli* were investigated by comparative analysis of their complete genomes, along with four draft genomes from these two species [16]. These differences can lead to identification of specific virulence factors among strains.

In the present study, we sequenced the genome of the rice-pathogenic *B. plantarii* ATCC 43733^T^ strain in order to compare its genome organization with that of *B. glumae* BGR1 and *B. gladioli* BSR3, and identify common and unique genes amongst these three *Burkholderia* rice pathogens. In addition, we compared the genome of these *Burkholderia* rice pathogens with the complete or draft genomes of other *Burkholderia* species, such as those found in different environmental habitats and those that are known to be pathogenic to animals and humans. Our comparative genome analysis demonstrates close relationships between the three rice pathogens and rice resulting in unique features of rice pathogenic *Burkholderia* species relative to other animal and human *Burkholderia* species.

## Results and discussion

### Genome sequencing and comparison

For comparative genome investigations of rice-pathogenic *Burkholderia* strains causing grain rot, sheath rot, or seedling blight, we examined the complete genome sequences from strains of *B. glumae* [13], *B. gladioli* [14], and *B. plantarii* (sequenced in the present study), along with publicly available complete or draft genomes from nine other *Burkholderia* strains (Table 1). The genomes ranged 4.9–9.0 Mbp in size, with a G + C content of 67.2–68.7%, and the number of predicted coded proteins was in the range of 4300–7400. Among the seven *Burkholderia* strains, the genome sizes were highly variable among and within species, although the G + C contents were very similar (Table 1). In the case of *B. glumae*, strain AU6208, harbored the smallest genome of ~4.9 Mbp, whereas strain BGR1 harbored the largest genome of ~7.2 Mbp. *B. glumae*, strain AU6208 was originally isolated from an infant patient with granulomatous disease and was pathogenic to rice. These findings suggest that *B. glumae* has evolved substantially, or has undergone rapid genome rearrangements or deletions, under different environments and hosts.

**Table 1:** **General features of genomes in** ***B. glumae*** **,** ***B. gladioli*** **, and** ***B. plantarii***

**Organ**	**Accession**	**Chromosome**	**Plasmid**	**Size**	**Gene**	**G + C content**	**Status**	**Origin**
	**Number**	**Number**	**Number**	**(bp)**	**Number**			
*B. plantarii* ATCC 43733^T^		2	1	8081051	6463	68.55	C^a^	Rice
*B. glumae* PG1	GCA_000835205	2	0	7896538	6561	68.77	C	
*B. glumae* BGR1	GCA_000022645	2	4	7284636	5773	67.93	C	Rice
*B. glumae* LMG 2196	GCA_000300755	ND^b^	ND	5814128	5173	67.23	UC^c^	Rice
*B. glumae* 3252-8	GCA_000365245	ND	ND	6190126	5996	67.23	UC	Rice
*B. glumae* AU6208	GCA_000300395	ND	ND	4957917	4361	67.31	UC	Human
*B. glumae* 336gr	GCA_000503955	ND	ND	6511812	6565	68.38	UC	Rice
*B. glumae* NCPPB3923	GCA_000801065	ND	ND	6663988	6067	68.29	UC	
*B. gladioli* BSR3	GCA_000194745	2	4	9052299	7410	67.4	C	Rice
*B. gladioli* 3848 s-5	GCA_000365265	ND	ND	7915969	7408	67.67	UC	Rice
*B. gladioli* UCD-UG_CHAPALOTE	GCA_000757585	ND	ND	8527129	7264	67.76	UC	Corn
*B. gladioli* NBRC 13700	GCA_000739755	ND	ND	8762606	7345	67.73	UC	

^a^Indicates “completed”.

^b^Indicates “not determinant”.

^c^Indicates “uncompleted”.

**Figure 1:**
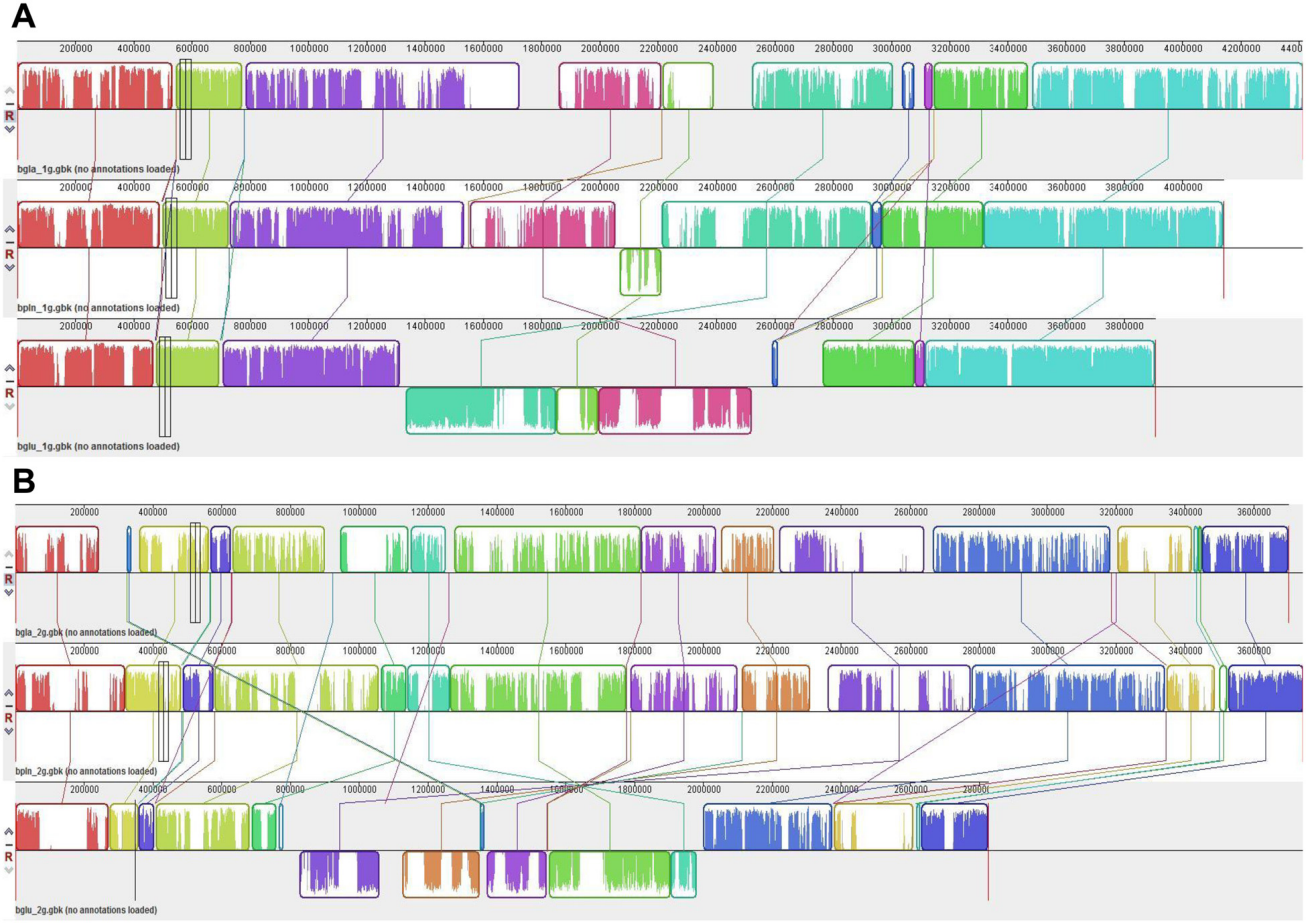
Multiple genome alignment for three *Burkholderia* strains: *Burkholderia glumae* BGR1, *B. gladioli* BSR3, and *B. plantarii* ATCC 43733^T^. The chromosome 1 **(A)** and chromosome 2 **(B)** sequences were aligned. The top, middle, and bottom sequences represent *B. gladioli* BSR3, *B. plantarii* ATCC 43733^T^, and *B. glumae* BGR1, respectively. Fine, colored lines represent rearrangements or inversions relative to the *B. plantarii* genome.

To better understand the interactions between rice-pathogenic *Burkholderia* species, comparative analysis was performed among the complete genome sequences of *B. glumae* BGR1, *B. gladioli* BSR3, and *B. plantarii* ATCC 43733^T^ (Table 2). Based on the Illumina HiSeq 2000 results, the genome of *B. plantarii* ATCC 43733^T^ was 8.08 Mbp and consisted of two chromosomes and one plasmid. Chromosome 1 contained 4,140,040 bp (68.4% G + C content) and 3,456 predicted coding sequences (CDS), while chromosome 2 contained 3,743,649 bp (69.1% G + C content) and 2,862 CDS; the plasmid bgla_1p contained 197,362 bp (62.4% G + C content) and 145 CDS. Although *B. glumae* BGR1 and *B. gladioli* BSR3 both have a genome comprising two chromosomes and four plasmids, the genome of *B. plantarii* ATCC 43733^T^ consists of two chromosomes and one plasmid. Multiple genome alignment for these three *Burkholderia* strains revealed a genome inversion in the middle of chromosomes 1 and 2 in *B. glumae* BGR1 when compared to the genomes of *B. gladioli* BSR3 and *B. plantarii* ATCC 43733^T^ (Figure 1A and B). The genome organization of *B. plantarii* ATCC 43733^T^ in the chromosome is much more similar to that of *B. gladioli* BSR3 than to that of *B. glumae* BGR1 (Figure 1A and B). MUMmer analysis and the size of the chromosome genome (Additional file 3: Figure S1 and Table 2) revealed a high number of genome deletions in chromosome 2 of *B. glumae* BGR1. Consistent with the observation of highly variable genome sizes in other *B. glumae* strains (Table 1), the genome of *B. glumae* appeared to be much more active than that of *B. gladioli* and *B. plantarii*.

**Table 2:** **Comparison of genome organization among the complete genome of three rice pathogenic** ***Burkholderia***

	***B. glumae*** **BGR1**	***B. gladioli*** **BSR3**	***B. plantarii*** **ATCC 43733** ^**T**^
Chr. 1	bglu_1g (3,906,507 bp, 3,495 genes)	bgla_1g (4,413,5616 bp, 3,964 genes)	bpln_1g (4,140,040 bp, 3,586 genes)
Chr. 2	bglu_2g (2,827,333 bp, 2,286 genes)	bgla_2g (3,700,833 bp, 3,006 genes)	bpln_2g (3,743,649 bp, 2,973 genes)
Plasmid 1	bglu_1p (133,579 bp, 144 genes)	bgla_1p (276,215 bp, 208 genes)	bpln_p (197,362 bp, 157 genes)
Plasmid 2	bglu_2p (141,792 bp, 121 genes)	bgla_2p (129,399 bp, 111 genes)	
Plasmid 3	bglu_3p (141,067 bp, 143 genes)	bgla_3p (128,650 bp, 96 genes)	
Plasmid 4	bglu_4p (134,369 bp, 115 genes)	bgla_4p (403,586 bp, 372 genes)	
Total	7,284,636 bp, 6,304 genes	9,052,299 bp, 7,757 genes	8,081,051 bp, 6,716 genes

### Genome comparison, pan-genome analysis, and core-genome analysis

To obtain better understanding of the genomic characteristics of *Burkholderia* rice pathogens as compared to a wider variety of *Burkholderia* strains, we conducted pan-genome analysis of 106 *Burkholderia* genomes (listed in Additional file 1: Table S1), including those from animal/human pathogens and those isolated from environmental habitats. Overall, 78,782 orthologs were identified in all organisms, constituting the pan-genome of these 106 *Burkholderia* strains (Additional file 4: Figure S2). Among the 78,782 pan-genome genes, 587 genes were highly conserved among the 106 *Burkholderia* genomes, constituting the core genome. Interestingly, the omission of the *B. glumae* LMG 2196 and *B. glumae* AU6208 strain genomes increased the number of genes in the core genome dramatically, to 848 genes. Thus, these two *B. glumae* strains may have rapidly evolved under the given environmental conditions.

**Figure 2:**
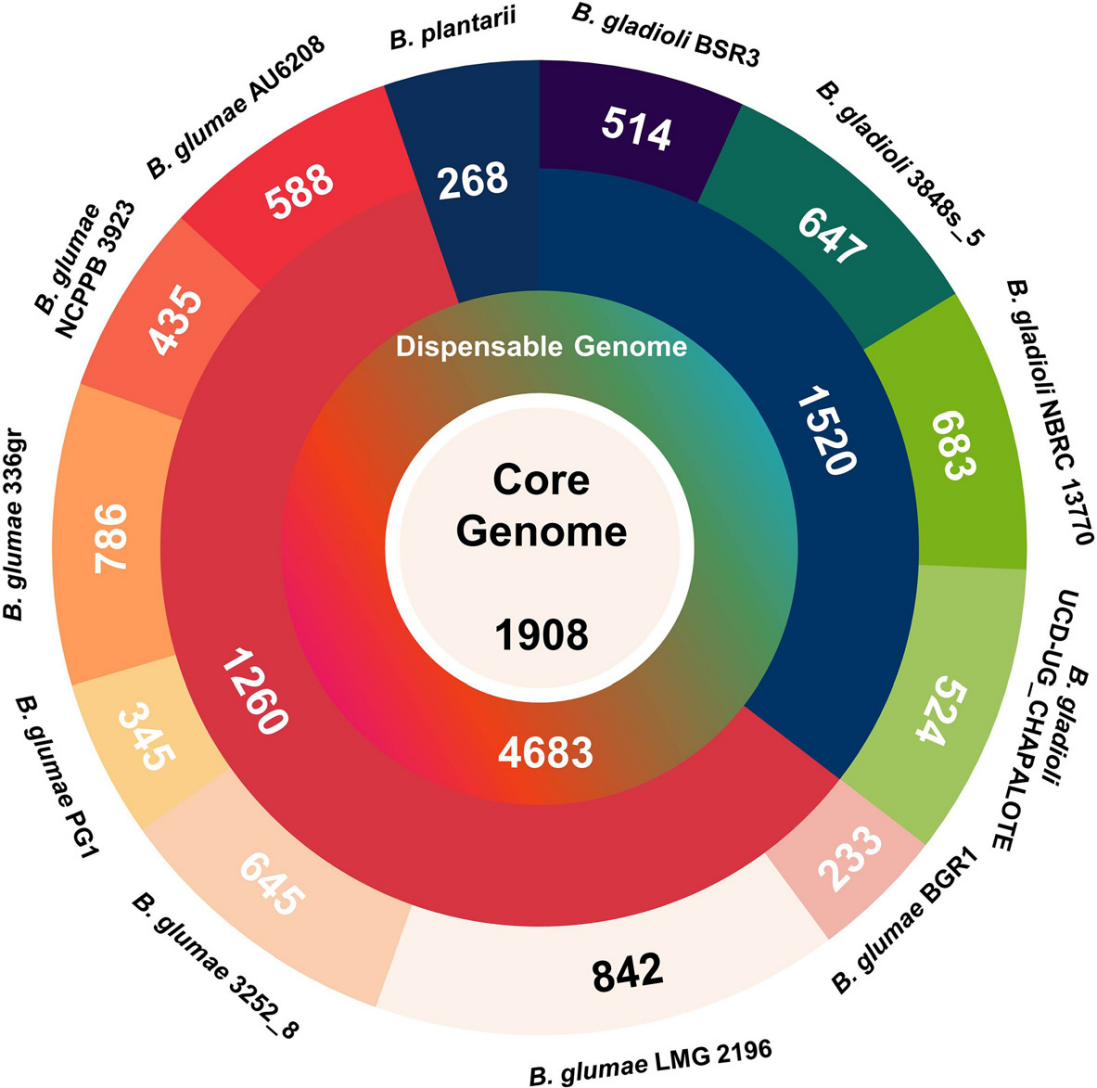
Pan-genome analysis of seven strains within *B. glumae*, *B. gladioli*, and *B. plantarii*. Core, dispensable, and strain-specific genomes are indicated in different colors.

The new genome sequence of *B. plantarii* ATCC 43733^T^ identified in the present study was combined with two full genomes of *B. gladioli* BSR3 and *B. glumae* BGR1, and four draft genomes in *B. glumae* and *B. gladioli* strains (Table 1) to identify a total of 12,758 orthologs that comprised the pan-genome of *B. gladioli*, *B. glumae*, and *B. plantarii*. Among these 12,758 genes, 1,908 genes were highly conserved and constituted the core genome of these seven *Burkholderia* strains (Figure 2). In addition, we identified 1,260 *B. glumae-*specific and 1,520 *B. gladioli*-specific genes. Among the seven *B. glumae* strains, the size of the strain-specific genome was ~340–840 genes (Figure 2), with the exception of *B. glumae* BGR1, which has only 233 strain-specific genes. As there were larger numbers of dispensable genes in *B. glumae* BGR1 than in other *B. glumae* strains, the *B. glumae* BGR1 genome could have stabilized or could be an original genome among these *B. glumae* strains.

### Bacterial secretion system

Diverse metabolites and proteins can be secreted into the environment or into host cells through bacterial secretion systems [17,18]. Each bacterial system has its own unique function, including conjugation, and these systems sometimes share functions such as pathogenicity. The 12 *Burkholderia* strains within *B. glumae*, *B. gladioli*, and *B. plantarii* species (listed in Table 1) have different numbers and types of secretion systems in their genomes. Genes involved in secretion-signal recognition particle (Sec-SRP) and twin arginine targeting (Tat) systems were highly conserved among all seven *Burkholderia* strains. The type III secretion system (T3SS) genes are also highly conserved in all 12 *Burkholderia* strains, except for deletion of *sctQ*, *sctR*, and *sctS* in the *B. glumae* LMG_2196 and AU6208 strains. Furthermore, with the exception of the partial sequence homology of *hrpW* in *B. gladioli* BRS3, the genes involved in the T3SS are nearly identical among *B. glumae* BGR1, *B. gladioli* BRS3, and *B. plantarii* ATCC 43733^T^ (Additioanl file 1: Table S2).

Evaluation of secretion system gene divergence revealed that all seven *Burkholderia* strains within the glumae group have one conserved type II secretion system (T2SS) on chromosome 1. However, *B. plantarii* ATCC 43733^T^ has an additional T2SS in chromosome 2, while two *B. gladioli* strains have two additional partial T2SS. Among the seven *Burkholderia* strains within the glumae group, only *B. glumae* BGR1, *B. glumae* AU6208, and *B. plantarii* ATCC 43733^T^ have a type I secretion system (T1SS), whereas only *B. gladioli* BSR3 and *B. plantarii* ATCC 43733^T^ have a type IV secretion system (T4SS) in their genomes. Thus, T1SS and T4SS show higher variability among the seven *Burkholderia* strains within the glumae group, as species-dependent total deletion of T1SS or T4SS was observed.

When compared to other genera, *Burkholderia* has a more diverse type VI secretion system (T6SS) with up to six T6SS gene clusters. Because the T6SS system can deliver bacterial proteins into both eukaryotic and prokaryotic cells, this secretion system is involved both in host pathogenesis and in anti-microbial mechanisms [19,20]. The T6SS apparatus structurally resembles an inverted bacteriophage tail that functions by injecting effector proteins directly into the cytosol of eukaryotic or bacterial cells. In particular, human- and animal-pathogenic *B. pseudomallei* and *B. mallei* have six T6SS gene clusters in their genome, four of which exist in both *B. pseudomallei* and *B. mallei* [21]. One T6SS is highly conserved among all 12 *Burkholderia* strains within the glumae group, which each harbor 2–4 T6SSs. Six T6SS groups can be classified in *Burkholderia* strains, based on the distribution of T6SS (Additional file 2: Table S3). T6SS_group1 was conserved in all genome-sequenced *Burkholderia* strains except for *B. xenovorans*, and was highly conserved among the seven *Burkholderia* strains within the glumae group. T6SS_group4 and T6SS_group5 were more specific to *B. glumae* or *B. plantarii* species: T6SS_group4 was only conserved among *B. glumae* and *B. ambifaria*; T6SS_group5 was only conserved among *B. glumae* and *B. plantarii*; and T6SS_group6 was only conserved among *B. glumae*, *B. graminis*, and *B. plantarii*. Different numbers of T6SS and unique T6SS in each species or strain indicate that T6SS could contribute to various inter-species interactions, including pathogen-host interactions and interactions with other microbes in the *Burkholderia* genus.

### QS systems

Bacterial QS is a form of cell-to-cell communication that uses chemical signaling between bacterial cells to regulate biological processes in response to environmental clues [22]. N-acylhomoserine lactone (AHL), the best known QS chemical signal, plays a key role in the regulatory circuit composed of a signal producer designated LuxI and a cognate receptor-regulatory protein designated LuxR [23]. *Burkholderia glumae* BGR1 QS uses a TofI-TofR circuit, similar to the LuxI-LuxR circuit, to regulate toxoflavin biosynthesis, flagella regulation, and detoxification of reactive oxygen species (ROS) [8-10]. Remarkably, *B. glumae* BGR1 QS protects stationary-phase cells from self-intoxication by altering cellular metabolism through the production of oxalate [24].

In this study, we surveyed AHL synthase and regulator in the genomes of 12 strains within *B. glumae*, *B. gladioli*, and *B. plantarii* species (listed in Table 1). Overall, 16 paired AHL synthase-regulator circuits were identified in 12 strains (Table 3). One paired AHL synthase-regulator circuit displayed high sequence homology in all 12 strains except for *B. gladioli* NBRC 13700. An additional paired AHL synthase-regulator circuit was found in the genome of *B. gladioli* BSR3, residing in the polyketide synthesis operon of the plasmid. Furthermore, *B. plantarii* ATCC 43733^T^ and *B. glumae* PG1 had two additional paired AHL synthase-regulator circuits; one AHL circuit (bpln_2g10770-bpln_2g10790 and AJK 49063.1-AJK 49065.1) was located close to genes involved in the urea/branched-chain amino acid, and the other AHL circuit (bpln_2g04430-bpln_2g04440 and AJK 48489.1-AJK 48490.1) resided near the genes involved in thiopurine biosynthesis.

**Table 3:** **Paired N-acylhomoserine lactone (AHL) synthase-regulator in** ***Burkholderia gladioli*** **,** ***B. glumae*** **, and** ***B. plantarii***

**Strain**	**Synthase**	**Regulator**	**(Putative** ^**a**^ **) Regulation**	**class**
*B. gladioli* BSR3	bgla_2g11050	bgla_2g11070	Toxoflavine synthesis	I
	bgla_1p1740	bgla_1p1760	(Putative) Polyketide synthesis	II
*B. glumae* BGR1	bglu_2g14490	bglu_2g14470	Toxoflavine synthesis	I
*B. plantarii* ATCC 43733^T^	bpln_2g10770	bpln_2g10790	(Putative) Urea/amino acid regulation	I
	bpln_1g07720	bpln_1g07790	Tropolon synthesis	III
	bpln_2g04430	bpln_2g04440	(Putative) Thiopurine/polymyxin	IV
*B. glumae* PG1	AJK49063.1	AJK49065.1	(Putative) Urea/amino acid regulation	I
	AJK45325.1	AJK45332.1	Tropolon synthesis	III
	AJK48489.1	AJK48490.1	(Putative) Thiopurine/polymyxin	IV
*B. gladioli* 3848 s-5	bgla3848_2451lmp	bgla3848_2453l		I
*B. gladioli* NBRC	ND	ND		
*B. gladioli* UCD	WP_036034986.1	WP_025097948.1		I
*B. glumae* 3252-8	bglu3252_0759lmp	bglu3252_0761l		I
*B. glumae* LMG 2196	BGLMG_03131	not predicted		I
*B. glumae* 336gr	WP_015877501.1	WP_015877499.1		I
*B. glumae* NCPPB	NCPPB3923_RS01185	NCPPB3923_RS01195		I
*B. glumae* AU6208	BGAU_02315	BGAU_02313		I

^a^Putative regulation is based on the location of synthase and regulator genes in the operon.

Without the AHL synthase pair, seven to twelve orphan AHL regulators existed in the genome of these 12 *Burkholderia* strains. Three orphan AHL regulators were highly conserved in all 12 *Burkholderia* strains. Twelve orphan AHL regulators were randomly distributed in the genome of *B. plantarii* ATCC. Overall, *B. plantarii* ATCC had the maximum number of AHL regulators among the 12 *Burkholderia* strains, suggesting that this strain synthesizes diverse auto-inducers and activates complicated regulatory systems in response to bacterial cell-to-cell communication.

### Toxin production

*Burkholderia* toxin is a key virulence factor responsible for diseases in plants. Toxoflavin is the most well-known phytopathogenic *Burkholderia* toxin produced by *B. glumae*, and is a host-nonspecific phytotoxin that is a very effective electron carrier and generates ROS such as hydrogen [8,10]. Genes involved in toxin biosynthesis were surveyed in 12 strains within *B. glumae*, *B. gladioli*, and *B. plantarii* species (listed in Table 1). Toxoflavin biosynthesis genes were distributed in all 12 *Burkholderia* strains except for *B. plantarii* ATCC 43733^T^ and *B. glumae* PG1 (Table 4). All *B. glumae* and *B. gladioli* strains harbored genes involved in the biosynthesis and transport of toxoflavin, except for a deletion of *toxI* in the genome of *B. glumae* AU6208. However, *B. plantarii* ATCC 43733^T^ only had the *toxJ* gene, a regulator of toxin biosynthesis.

**Table 4:** **Genes involved in toxoflavin biosynthesis in twelve strains within** ***B. glumae*** **,** ***B. gladioli*** **, and** ***B. plantarii***

**Gene**	**BGR1** ^**a**^	**bgluLMG** ^**b**^	**bglu3252** ^**c**^	**bgluAU** ^**d**^	**Bglu336gr**	**bgluNCPPB**	**BSR3** ^**e**^	**bgla3848** ^**f**^	**bglaNBRC**	**bglaUCD**	**bpln** ^**g**^	**bgluPG1**
*toxJ*	bglu_2g06330	831/831^h^	bglu3252_4487l	787/789	WP_012733464.1	NCPPB3923_RS00965	bgla_2g09030	bgla3848_0587lmp	WP_025099873.1	WP_036035589.1	bpln_2g08940	AJK48890.1
*toxI*	bglu_2g06350	BGLMG_03249	bglu3252_6550lmp	ND^i^	381/381	NCPPB3923_RS00955	bgla_1g04520	1125/1128	WP_036052885.1	WP_036038556.1	bpln_2g04220	AJK47580.1
*toxH*	bglu_2g06360	3092/3094	bglu3252_4548lmp	3086/3093	976/976	NCPPB3923_RS00950	bgla_1g04530	bgla3848_4122lmp	WP_036048419.1	WP_036030576.1	ND	ND
*toxG*	bglu_2g06370	BGLMG_03246	bglu3252_4547lmp	BGAU_04306	WP_012733468.1	NCPPB3923_RS00945	bgla_1g04540	bgla3848_4123lmp	WP_036048416.1	WP_036030574.1	ND	ND
*toxF*	bglu_2g06380	BGLMG_02566	bglu3252_2104lmp	BGAU_04308	WP_012733469.1	NCPPB3923_RS00940	bgla_1g04550	bgla3848_4124lmp	WP_036048413.1	WP_036030571.1	ND	ND
*toxR*	bglu_2g06390	BGLMG_02565	bglu3252_2105lmp	BGAU_04309	WP_012733470.1	NCPPB3923_RS00935	bgla_1g04560	bgla3848_4125lmp	WP_025100566.1	WP_036030568.1	ND	ND
*toxA*	bglu_2g06400	BGLMG_02564	bglu3252_2107lp	BGAU_04310	WP_012733471.1	NCPPB3923_RS00930	bgla_1g04570	bgla3848_4128lp	WP_036048410.1	WP_036030565.1	ND	ND
*toxB*	bglu_2g06410	BGLMG_02563	bglu3252_2108lmp	403/403	260/260	NCPPB3923_RS00925	bgla_1g04580	bgla3848_4129lmp	WP_013696509.1	WP_013696509.1	ND	ND
*toxC*	bglu_2g06420	BGLMG_02562	bglu3252_2109lmp	749/751	572/572	NCPPB3923_RS00920	bgla_1g04590	bgla3848_4130lmp	WP_036048408.1	WP_036030560.1	ND	ND
*toxD*	bglu_2g06430	BGLMG_02561	bglu3252_2110lmp	BGAU_03159	WP_012733474.1	NCPPB3923_RS00915	bgla_1g04600	bgla3848_4131lmp	WP_036048407.1	WP_036030557.1	ND	ND
*toxE*	bglu_2g06440	BGLMG_02560	1141/1147	BGAU_03158	WP_035978132.1	NCPPB3923_RS00910	bgla_1g04610	bgla3848_4132lmp	WP_036052884.1	WP_036030777.1	ND	ND

^a^Indicates “*B. glumae* BGR1”.

^b^Indicates “*B. glumae* LMG 2196”.

^c^Indicates “*B. glumae* 3252-8”.

^d^Indicates “*B. glumae* AU6208”.

^e^Indicates “*B. gladioli* BSR3”.

^f^Indicates “*B. gladioli*3848s-5”.

^g^ Indicates “*B. plantarii* ATCC 43733^T^”.

^h^Represents identities of nucleotide sequences.

^i^Indicates “not detected in the genome”.

Instead of producing toxoflavin, *B. plantarii* is known to produce tropolone as a phytotoxin and as a virulence factor causing seedling blight. Rice seedlings exposed to tropolone typically exhibit symptoms similar to those of *B. plantarii*-mediated rice seedling blight [25]. When we surveyed all publicly available *Burkholderia* strain genomes, the genes involved in tropolone biosynthesis were only identified in the genome of *B. plantarii* ATCC 43733^T^ and *B. glumae* PG1 (Additional file 1: Table S4). Interestingly, one paired AHL synthase-regulator circuit (bpln_1g07720-bpln_1g07790 and AJK 45325.1-AJK 45332.1) resided within the tropolone biosynthesis operon. This indicates that the regulation of tropolone biosynthesis may be dependent on bacterial cell-to-cell communication in a manner similar to that of the paired AHL circuit (bglu_2g14490-bpln_2g14470) in *B. glumae* BGR1, which regulates toxoflavin biosynthesis according to bacterial cell density [10], although these AHL circuit genes are not present in the toxoflavin biosynthesis operon.

Genes involved in rhizotoxin biosynthesis were also identified in the genome of *B. plantarii* ATCC 43733^T^. Rhizotoxin is an antimitotic agent with antitumor activity [26], isolated from a pathogenic plant fungus (*Rhizopus microsporus*). Rhizotoxin also causes rice seedling blight that results in the same symptoms as seedlings treated with tropolone. Genes involved in rhizotoxin biosynthesis have also been identified in several strains of bacteria, including *Xanthomonas oryzae* pv. *oryzae* KACC10331 *B. JYP251*, *B. phymatum*, *B. phenoliruptrix , and B. glumae* PG1 (Additional file 1: Table S5).

### Virulence-related enzymes

Genes encoding polygalacturonases, cellulases, lipases and proteases are major virulence factors in diverse pathogenic bacteria. These enzymes are related to the virulence and their regulation in *B. glumae* has been comprehensively summarized [7]. The characteristics, regulation, and virulence function of polygalacturonases in *B. glumae* was intensively investigated and *pehA* and *pehB* encoding two isoforms of polygalacturonases, have been discovered discovered [27]. The *pehA* locus was mainly distributed in *B. glumae* strains, whereas the *pehB* locus was detected in all *B. glumae*, *B. gladioli*, and *B. plantarii* strains (Additional file 2: Table S7). The roles of lipases have been studied, not only in plant pathogenic strains but also in human pathogenic *Burkholderia* strains with respect to the virulence [28,29]. The gene encoding the lipase LipA was detected in all *B. glumae*, *B. gladioli*, and *B. plantarii* strains except for *B. glumae* AU6208. These virulence-related enzymes in the 12 *Burkholderia* strains are summarized in Additional file 2: Table S7.

### Clustered regularly interspaced short palindromic repeats (CRISPR)-CRISPR-associated protein (Cas)

The CRISPR-Cas system is a bacterial immune system that protects bacteria from invading viruses and transferring plasmids [30,31]. Recent studies have indicated that the CRISPR-Cas system acts as a barrier to horizontal gene transfer and as a modulator of gene expression [32]. The CRISPR-Cas immune system blocks stable entry of foreign nucleic acids in three common steps: adaptation, CRISPR RNA (crRNA) biogenesis, and targeting [33,34]. During adaptation, viral or plasmid challenge stimulates the incorporation of short (24–48 nucleotide) invader-derived sequences between equally short DNA repeats found in the CRISPR locus [33,35]. These unique sequences, which are known as spacers, primarily match viruses and other mobile genetic elements [36].

**Figure 3:**
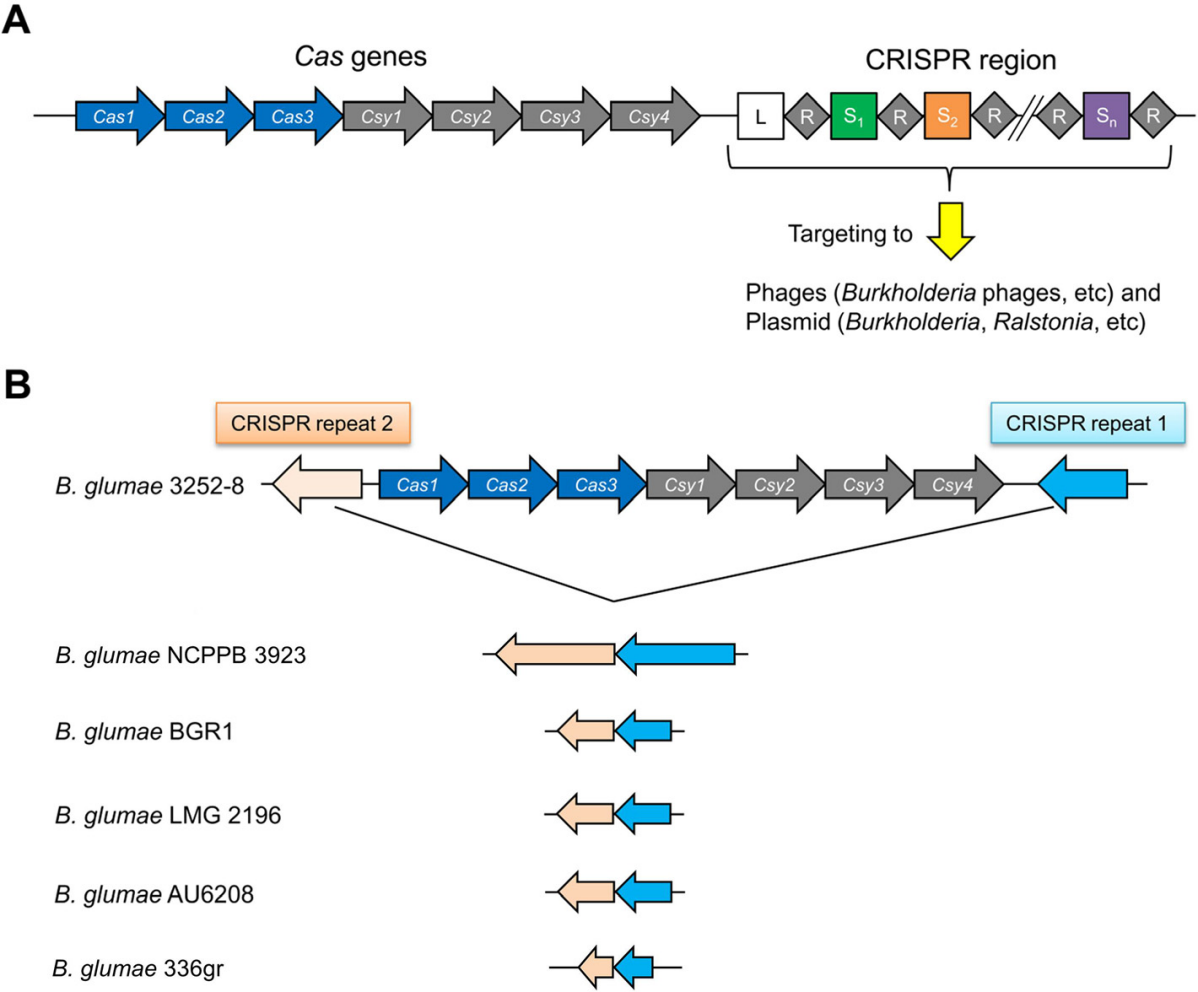
Diagram of the clustered regularly interspaced short palindromic repeats (CRISPR) with CRISPR associated proteins (Cas) system in *Burkholderia* species. **(A)** CRISPR-Cas in *B. plantarii* ATCC 43733^T^. R, S represent the CRISPR repeat and CRISPR spacer, respectively. **(B)** CRISPR-Cas in *B. glumae* 3252*–*8.

We surveyed the CRISPR-Cas system in 106 *Burkholderia* genomes (listed in Additional file 1: Table S1). Remarkably, two *B. plantarii* ATCC 43733^T^*, B. gladioli* USD UG_CHAPALOTE, *B. glumae* PG1, and *B. glumae* 3252–8 strains have one CRISPR-Cas system. The other eight strains in the *B. glumae* and *B. gladioli* species have only the CRISPR motif without Cas proteins. However, no clear CRIPSR motif was identified in pathogenic-animal and human *Burkholderia* strains. The CRIPSR-Cas system in *B. plantarii* ATCC 43733^T^ had an internal stop codon in the middle of the *cas1* gene, leading to two separate Cas1; thus, the *cas* operon was composed of Cas1 (bpln_1g17440), Cas2 (bpln_1g17450), Cas3 (bpln_1g17460), Csy1 (bpln_1g17470), Csy2 (bpln_1g17480), Csy3 (bpln_1g17490), and Csy4 (bpln_1g17500) (Figure 3A). Among the 12 strains, *B. gladioli, B. glumae,* and *B. plantarii* species had four types of CRIPSR repeats, with the *B. plantarii* ATCC 43733^T^ and *B. glumae* 3252–8 strains sharing the common CRIPSR repeat (TTTCTAAGCTGCCTACACGGCAGCGAAC). Interestingly, *B. glumae* 3252–8 contained the *cas* operon between two CRIPSR repeats. Other five *B. glumae* strains had one or two CRISPR repeats without the *cas* operon (Figure 3B). These findings suggest that the *cas* operon was present in *B. glumae,* but was subsequently deleted in most *B. glumae*. Deletion events of the *cas* operon may have occurred in many *Burkholderia* strains; thus, we were only able to identify the *cas* operon in *B. plantarii* ATCC 43733^T^*, B. glaidioli* USD UG_CHAPALOTE*, B. glumae* PG1, and *B. glumae* 3252–8 from the genome sequences of over 100 *Burkholderia* strains.

We analyzed CRISPR targets, based on sequences of the CRISPR spacers in *B. plantarii* ATCC 43733^T^ and *B. glumae* 3252–8, using Viroblast (http://indra.mullins.microbiol.washington.edu/viroblast/viroblast.php) or BLAST plasmid searches. The spacer/targeting sequences revealed diverse phage targets, including *Burkholderia* phages*,* other bacterial phages, and various types of plasmids (Additional file 2: Table S6). Interestingly, the CRISPR repeat (TTTCTAAGCTGCCTACACGGCAGCGAAC) common to both *B. plantarii* ATCC 43733^T^ and *B. glumae* 3252–8 harbored the largest number of spacers. Specifically, there were 21 spacers in *B. plantarii* ATCC 43733^T^ and 12 spacers in *B. glumae* 3252–8. Three of 21 spacers in *B. plantarii* ATCC 43733^T^ targeted several *Burkholderia* phages, including phage BcepC6B, phage KS14, and phage KL3, as well as plasmids of *B. ambifaria* MC40-6*, B. cenocepacia, B. multivorans,* and *B. vietnamiensis* with high sequence identities (Additional file 2: Table S6). However, 2 spacers among 12 in *B. glumae* 3252–8 targeted different types of bacteriophages, including *Murine adenovirus* 2 and *Saccharopolyspora erythraea* NRRL2338 with high sequence identities, but did not target bacterial plasmids.

## Conclusions

The complete genome sequencing of *B. plantarii* ATCC 43733^T^ performed in this study, and publicly available genomes of *B. glumae* BGR1 and *B. gladioli* BSR3, enabled comprehensive comparative genome analyses among three rice-pathogenic *Burkholderia* species responsible for tissue rotting and seedling blight. The genome organization and chromosome structure in *B. plantarii* ATCC 43733^T^ are more similar to those of *B. gladioli* BSR3, which is consistent with the finding that *B. plantarii* ATCC 43733^T^ and *B. gladioli* BSR3 are closely related based on 16S rRNA sequences. Genome analyses of interesting gene clusters such as secretion system genes, toxin production genes, bacterial QS genes, and CRISPR-mediated immune system genes indicated that *B. plantarii* ATCC 43733^T^ has more diverse gene pairs in the QS-mediated AHL synthase-receptor circuit and in unique bacterial toxins such as tropolone and rhizotoxin. Interestingly, only the genomes of *B. plantarii* ATCC 43733^T^*, B. glaidioli* USD UG_CHAPALOTE*, B. glumae* PG1*,* and *B. glumae* 3252*–*8 harbored complete CRISPR-Cas systems, among all genome-sequenced for *Burkholderia* strains. Based on genome organization and toxin production, *B. glumae* PG1 was more closely related to *B. plantarii* ATCC 43733^T^ than to the other *B. glumae* strains. Better knowledge of the variability and specificities of *Burkholderia* organisms could contribute to an understanding of their capacity to adapt to different environments, as well as their unique interactions with the host during pathogenesis.

## Methods

### Genome sequencing of *B. plantarii* ATCC 43733^T^

Whole-genome shotgun DNA sequencing of *B. plantarii* ATCC 43733^T^ was conducted using an Illumina HiSeq 2000. In total, 200,106,179 paired-end reads were analyzed. The genomic shotgun sequence data were assembled with an ABySS [37] assembler, and contig ordering was confirmed by the 95,596 paired-end reads obtained from the 8-kb insert library using the Roche/454 pyrosequencing method on a Genome Sequencer FLX system. Gaps among contigs were closed by a combination of primer walking on gap-spanning clones and direct sequencing of combinatorial PCR products.

### Gene annotation of *B. plantarii* ATCC 43733^T^

Coding genes and pseudogenes across the genome were predicted using Glimmer [38], GeneMarkHMM [39], and Prodigal [40], and were annotated by comparison with the NCBI-NR database [41]. Our annotation results were verified using Artemis [42].

### Nucleotide sequence accession number of *B. plantarii* ATCC 43733^T^

The sequences of *B. plantarii* ATCC 43733^T^ chromosome 1, chromosome 2, and plasmid genome have been deposited in GenBank under accession numbers CP007212, CP007212, and CP007212, respectively.

### Comparative and pan-genome analysis

A total of 111 *Burkholderia* genome sequences (with 37 complete and 74 draft genome sequences) were downloaded from NCBI. 16S ribosomal RNA sequences were used to construct a phylogenetic tree using the unweighted pair group method with arithmetic mean (UPGMA) with MEGA6 software. Based on phylogenetic analysis, we divided *Burkholderia* species into a glumae group, cepacia group, mallei group, and outgroup (Additional file 5: Figure S5). We discarded five *Burkholderia* species, including *B. rhizoxinica*, because these species have higher genome variation owing to occupying ecological niches such as symbiosis. Overall, 12, 27, 49, and 18 species belonged to the glumae group, cepacia group, mallei group, and outgroup, respectively (Additional file 1: Table S1). For annotation of the unfinished genome and to make CDS prediction easier, all scaffolds for each strain were linked into a pseudochromosome according to the coordinates of ATCC_9150 with a piece of a random sequence. The scaffold linker (NNN NNC ATT CCA TTC ATT AAT TAA TTA ATG AAT GAA TGN NNN N) contains stop and start codons in all six frames, so it could prevent the protein-coding genes from extending from one scaffold to the next [43]. Pan-genome analysis was performed on a larger dataset of these 106 *Burkholderia* genomes using the GeneFamily method in the pan-genome analysis pipeline [44]. All proteins were filtered with the criteria of 50% coverage, 50% identity, and a 1.0 × e^−10^ e-value, and ortholog clusters were generated using MCL software [45].

### CRISPR-Cas system

The CRISPRs Finder tool (http://crispr.u-psud.fr/Server/) was used to search for CRISPR direct repeats and spacers in the sequenced *Burkholderia* strains, which were then compared to JGI (http://www.jgi.doe.gov) analysis results. The CRISPR repeats were aligned in the genome and the sequences and locations of spacers were identified. We used Viroblast (http://indra.mullins.microbiol.washington.edu/viroblast/viroblast.php) and local BLAST analysis against NCBI plasmid genomes (ftp://ftp.ncbi.nlm.nih.gov/genomes/Plasmids/) to identify the targets of the spacers.

### Availability of supporting data

All supporting data are included within the article and its additional files.

## Additional files

Additional file 1: Table S1. Genome information regarding 106 *Burkholderia* species used for pan-genome analysis. **Table S2.** Genes involved in Type III secretion among *Burkholderia glumae* BGR1, *B. gladioli* BSR3, and *B. plantarii* ATCC 43733^T^. **Table S4.** Genes involved in tropolone biosynthesis in *B. plantarii* ATCC 43733^T^. **Table S5.** Genes involved in rhizotoxin biosynthesis among bacteria strains. Additional file 2: Table S3. The type VI secretion system (T6SS) in seven strains within *B. glumae, B. gladioli*, and *B. plantarii* species. **Table S6.** Lists of CRISPR target viruses or plasmids based on spacer sequences. **Table S7.** Distribution of genes encoding polygalacturonases, celluases, lipases, and proteases that are involved in virulence among *Burkholderia* strains. Additional file 3: Figure S1. MUMmer analysis of each chromosome between *Burkholderia glumae* BGR1, *B. gladioli* BSR3, and *B. plantarii* ATCC 43733^T^. Additional file 4: Figure S2. Pan-genome and core-genome analysis based on 106 genomes of *Burkholderia* strains (listed in Additional file 1: Table S1). The blue box, violet box, green box, and pink box represent the glumae group, cepacia group, mallei group, and outgroup, respectively. Each group is designated in Additional file 1: Table S1 and Additional file 5: Figure S3. Additional file 5: Figure S3. Phylogenetic tree of 106 *Burkholderia* species based on 16S rRNA sequences.

## Abbreviations

Bcc: *Burkholderia cepacia* complex
CRISPR-Cas: Clustered regularly interspaced short palindromic repeats-CRISPR associated proteins
CDS: Coding sequences
Sec-SRP: Secretion-signal recognition particle
Tat: Twin arginine targeting
T1SS: Type I secretion system
T2SS: Type II secretion system
T3SS: Type III secretion system
T4SS: Type IV secretion system
T6SS: Type VI secretion system
AHL: N-acylhomoserine lactone
ROS: Reactive oxygen species.

## References

[1] Compant S, Nowak J, Coenye T, Clément C, Ait Barka E (2008). Diversity and occurrence of *Burkholderia* spp. in the natural environment. FEMS Microbiol Rev.

[2] Vial L, Groleau M-C, Dekimpe V, Déziel E (2007). *Burkholderia* diversity and versatility: an inventory of the extracellular products. J Microbiol Biotechnol.

[3] Valvano MA, Keith KE, Cardona ST (2005). Survival and persistence of opportunistic *Burkholderia* species in host cells. Curr Opin Microbiol.

[4] Ait Barka E, Nowak J, Clément C (2006). Enhancement of chilling resistance of inoculated grapevine plantlets with a plant growth-promoting rhizobacterium, *Burkholderia phytofirmans* strain PsJN. Appl Environ Microbiol.

[5] Compant S, Reiter B, Sessitsch A, Nowak J, Clément C, Ait Barka E (2005). Endophytic colonization of *Vitis vinifera* L. by plant growth-promoting bacterium *Burkholderia* sp. strain PsJN. Appl Environ Microbiol.

[6] Reis VM, Estrada-de los Santos P, Tenorio-Salgado S, Vogel J, Stoffels M, Guyon S (2004). *Burkholderia tropica* sp. nov., a novel nitrogen-fixing, plant-associated bacterium. Int J Syst Evol Microbiol.

[7] Ham JH, Melanson RA, Rush MC (2011). *Burkholderia glumae*: next major pathogen of rice?. Mol Plant Pathol.

[8] Chun H, Choi O, Goo E, Kim N, Kim H, Kang Y (2009). The quorum sensing-dependent gene *katG* of *Burkholderia glumae* is important for protection from visible light. J Bacteriol.

[9] Kim J, Kang Y, Choi O, Jeong Y, Jeong J-E, Lim JY (2007). Regulation of polar flagellum genes is mediated by quorum sensing and FlhDC in *Burkholderia glumae*. Mol Microbiol.

[10] Kim J, Kim J-G, Kang Y, Jang JY, Jog GJ, Lim JY (2004). Quorum sensing and the LysR-type transcriptional activator ToxR regulate toxoflavin biosynthesis and transport in *Burkholderia glumae*. Mol Microbiol.

[11] Nandakumar R, Shahjahan AKM, Yuan XL, Dickstein ER, Groth DE, Clark CA (2009). *Burkholderia glumae* and *B. gladioli* cause bacterial panicle blight in rice in the southern united states. Plant Dis.

[12] Solis R, Bertani I, Degrassi G, Devescovi G, Venturi V (2006). Involvement of quorum sensing and RpoS in rice seedling blight caused by *Burkholderia plantarii*. FEMS Microbiol Lett.

[13] Lim J, Lee T-H, Nahm BH, Choi YD, Kim M, Hwang I (2009). Complete genome sequence of *Burkholderia glumae* BGR1. J Bacteriol.

[14] Seo Y-S, Lim J, Choi B-S, Kim H, Goo E, Lee B (2011). Complete genome sequence of *Burkholderia gladioli* BSR3. J Bacteriol.

[15] Francis F, Kim J, Ramaraj T, Farmer A, Rush MC, Ham JH (2013). Comparative genomic analysis of two *Burkholderia glumae* strains from different geographic origins reveals a high degree of plasticity in genome structure associated with genomic islands. Mol Genet Genomics.

[16] Fory PA, Triplett L, Ballen C, Abello JF, Duitama J, Aricapa MG (2014). Comparative analysis of two emerging rice seed bacterial pathogens. Phytopathology.

[17] Hayes CS, Aoki SK, Low DA (2010). Bacterial contact-dependent delivery systems. Annu Rev Genet.

[18] Thanassi DG, Bliska JB, Christie PJ (2012). Surface organelles assembled by secretion systems of Gram-negative bacteria: diversity in structure and function. FEMS Microbiol Rev.

[19] Ho BT, Dong TG, Mekalanos JJ (2014). A view to a kill: the bacterial type VI secretion system. Cell Host Microbe.

[20] Kapitein N, Mogk A (2013). Deadly syringes: type VI secretion system activities in pathogenicity and interbacterial competition. Curr Opin Microbiol.

[21] Burtnick MN, Brett PJ, Harding SV, Ngugi SA, Ribot WJ, Chantratita N (2011). The cluster 1 type VI secretion system is a major virulence determinant in *Burkholderia pseudomallei*. Infect Immun.

[22] Chen J, Xie J (2011). Role and regulation of bacterial LuxR-like regulators. J Cell Biochem.

[23] Fuqua WC, Winans SC, Greenberg EP (1994). Quorum sensing in bacteria: the LuxR-LuxI family of cell density-responsive transcriptional regulators. J Bacteriol.

[24] Goo E, Majerczyk CD, An JH, Chandler JR, Seo Y-S, Ham H (2012). Bacterial quorum sensing, cooperativity, and anticipation of stationary-phase stress. Proc Natl Acad Sci U S A.

[25] Azegami K, Nishiyama K, Watanabe Y, Suzuki T, Yoshida M (1985). Tropolone as a root growth-inhibitor produced by a plant pathogenic *Pseudomonas* sp. causing seedling blight of rice. Ann Phytopathol Soc Japan.

[26] Tsuruo T, Oh-hara T, Iida H, Tsukagoshi S, Sato Z, Matsuda I (1986). Rhizoxin, a macrocyclic lactone antibiotic, as a new antitumor agent against human and murine tumor cells and their vincristine-resistant sublines. Cancer Res.

[27] Degrassi G, Devescovi G, Kim J, Hwang I, Venturi V (2008). Identification, characterization and regulation of two secreted polygalacturonases of the emerging rice pathogen *Burkholderia glumae*. FEMS Microbiol Ecol.

[28] Devescovi G, Bigirimana J, Degrassi G, Cabrio L, LiPuma JJ, Kim J (2007). Involvement of a quorum-sensing-regulated lipase secreted by a clinical isolate of *Burkholderia glumae* in severe disease symptoms in rice. Appl Environ Microbiol.

[29] Mullen T, Markey K, Murphy P, McClean S, Callaghan M (2007). Role of lipase in *Burkholderia cepacia* complex (Bcc) invasion of lung epithelial cells. Eur J Clin Microbiol Infect Dis.

[30] Louwen R, Staals RHJ, Endtz HP, van Baarlen P, van der Oost J (2014). The role of CRISPR-Cas systems in virulence of pathogenic bacteria. Microbiol Mol Biol Rev.

[31] Koonin EV, Makarova KS (2013). CRISPR-Cas: evolution of an RNA-based adaptive immunity system in prokaryotes. RNA Biol.

[32] Hatoum-Aslan A, Marraffini LA (2014). Impact of CRISPR immunity on the emergence and virulence of bacterial pathogens. Curr Opin Microbiol.

[33] Gasiunas G, Sinkunas T, Siksnys V (2014). Molecular mechanisms of CRISPR-mediated microbial immunity. Cell Mol Life Sci.

[34] Makarova KS, Haft DH, Barrangou R, Brouns SJJ, Charpentier E, Horvath P (2011). Evolution and classification of the CRISPR-Cas systems. Nat Rev Microbiol.

[35] Barrangou R, Fremaux C, Deveau H, Richards M, Boyaval P, Moineau S (2007). CRISPR provides acquired resistance against viruses in prokaryotes. Science.

[36] Stern A, Keren L, Wurtzel O, Amitai G, Sorek R (2010). Self-targeting by CRISPR: gene regulation or autoimmunity?. Trends Genet.

[37] Simpson JT, Wong K, Jackman SD, Schein JE, Jones SJM, Birol I (2009). ABySS: a parallel assembler for short read sequence data. Genome Res.

[38] Delcher AL, Harmon D, Kasif S, White O, Salzberg SL (1999). Improved microbial gene identification with GLIMMER. Nucleic Acids Res.

[39] Lukashin AV, Borodovsky M (1998). GeneMark.hmm: new solutions for gene finding. Nucleic Acids Res.

[40] Hyatt D, Chen G-L, Locascio PF, Land ML, Larimer FW, Hauser LJ (2010). Prodigal: prokaryotic gene recognition and translation initiation site identification. BMC Bioinformatics.

[41] Benson DA, Karsch-Mizrachi I, Lipman DJ, Ostell J, Wheeler DL (2008). GenBank. Nucleic Acids Res.

[42] Rutherford K, Parkhill J, Crook J, Horsnell T, Rice P, Rajandream MA (2000). Artemis: sequence visualization and annotation. Bioinformatics.

[43] Liang W, Zhao Y, Chen C, Cui X, Yu J, Xiao J (2012). Pan-genomic analysis provides insights into the genomic variation and evolution of *Salmonella* Paratyphi A. PLoS One.

[44] Zhao Y, Wu J, Yang J, Sun S, Xiao J, Yu J (2012). PGAP: pan-genomes analysis pipeline. Bioinformatics.

[45] Van Dongen S. A Cluster Algorithm for Graphs. PhD thesis, National Research Institute for Mathematics and Computer Science in the Netherlands, 2000.

