# Supplementary material for: Comparative genome analysis of rice-pathogenic Burkholderia provides insight into capacity to adapt to different environments and hosts
Source: BMC Genomics. 2015 May 6;16(1):349. doi: 10.1186/s12864-015-1558-5 (PMC4422320; doi:10.1186/s12864-015-1558-5)
Supplement: Additional file 1: Table S1. — Genome information regarding 106 Burkholderia species used for pan-genome analysis. Table S2. Genes involved in Type III secretion among Burkholderia glumae BGR1, B. gladioli BSR3, and B. plantarii ATCC 43733T. Table S4. Genes involved in tropolone biosynthesis in B. plantarii ATCC 43733T. Table S5. Genes involved in rhizotoxin biosynthesis among bacteria strains. [file 12864_2015_1558_MOESM1_ESM.docx]

**Table S1. Genome information regarding 106 *Burkholderia* species used for pan-genome analysis.**

| Group | Genome ID | Number of  chromosomes | Number of  Plasmids | Size | Number of  genes | G+C  content | Status | Annotation |
| --- | --- | --- | --- | --- | --- | --- | --- | --- |
| glumae | B_plantarii_uid | 2 | 1 | 8081051 | 6463 | 68.55 | C^a^ | registered* |
|  | B_glumae_BGR1_uid59397  B_glumae_PG1  B_glumae_336gr  B_glumae_NCPPB3923 | 2  2  ND  ND | 4  0  ND  ND | 7284636  7896538  6511812  6663988 | 5773  6561  6565  6067 | 67.93  68.77  68.38  68.29 | C  C  UC  UC | registered  registered  registered  registered |
|  | B_glumae_LMG_2196_uid199802 | ND^b^ | ND | 5814128 | 5173 | 67.23 | UC^c^ | local annotation |
|  | B_glumae_3252_8_uid199978 | ND | ND | 6190126 | 5996 | 67.23 | UC | JGI |
|  | B_glumae_AU6208_uid199798 | ND | ND | 4957917 | 4361 | 67.31 | UC | local annotation |
|  | B_gladioli_BSR3_uid66301 | 2 | 4 | 9052299 | 7410 | 67.4 | C | registered |
|  | B_gladioli_3848s_5_uid199977  B_gladioli_UCD-UG_CHAPALOTE  B_gladioli_NBRC_13700 | ND  ND  ND | ND  ND  ND | 7915969  8527129  8762606 | 7408  7264  7345 | 67.67  67.76  67.73 | UC  UC  UC | JGI  registered  registered |
| cepacia | B_cepacia_GG4_uid173858 | 2 | 0 | 6467321 | 5825 | 66.68 | C | registered |
|  | B_cenocepacia_AU_1054_uid58371 | 3 | 0 | 7279116 | 6477 | 66.93 | C | registered |
|  | B_cenocepacia_BC7_uid188101 | ND | ND | 7955909 | 8087 | 66.64 | UC | registered |
|  | B_cenocepacia_H111_uid180971 | ND | ND | 7680409 | 7258 | 67.34 | UC | registered |
|  | B_cenocepacia_HI2424_uid58369 | 3 | 1 | 7702840 | 6919 | 66.79 | C | registered |
|  | B_cenocepacia_J2315_uid57953 | 3 | 1 | 8055782 | 7107 | 66.9 | C | registered |
|  | B_cenocepacia_K56_2Valvano_uid188102 | ND | ND | 7674858 | 7707 | 66.81 | UC | registered |
|  | B_cenocepacia_MC0_3_uid58769 | 3 | 0 | 7971389 | 7008 | 66.6 | C | registered |
|  | B_cenocepacia_PC184_uid54353 | ND | ND | 7035252 | 5535 | 66.82 | UC | registered |
|  | B_ambifaria_AMMD_uid58303 | 3 | 1 | 7528567 | 6610 | 66.77 | C | registered |
|  | B_ambifaria_IOP40_10_uid54913 | ND | ND | 7716999 | 6923 | 66.22 | UC | registered |
|  | B_ambifaria_MC40_6_uid58701 | 3 | 0 | 7642536 | 6697 | 66.39 | C | registered |
|  | B_ambifaria_MEX_5_uid54911 | ND | ND | 7885782 | 7124 | 65.83 | UC | registered |
|  | B_multivorans_ATCC_17616_uid58697 | 3 | 0 | 7008622 | 6258 | 66.69 | C | registered |
|  | B_multivorans_ATCC_17616_uid58909 | 3 | 0 | 7008810 | 6111 | 66.69 | C | registered |
|  | B_multivorans_ATCC_BAA_247_uid180862 | ND | ND | 6222631 | 6297 | 67 | UC | registered |
|  | B_multivorans_CGD1_uid55347 | ND | ND | 6623140 | 6572 | 66.98 | UC | registered |
|  | B_multivorans_CGD2M_uid55365 | ND | ND | 6565486 | 6646 | 66.99 | UC | registered |
|  | B_multivorans_CGD2_uid55349 | ND | ND | 6555695 | 6653 | 66.99 | UC | registered |
|  | B_multivorans_uid180863 | ND | ND | 6520774 | 6633 | 66.76 | UC | registered |
|  | B_383_uid58073 | 3 | 0 | 8676277 | 7716 | 66.27 | C | registered |
|  | B_KJ006_uid165871 | 3 | 1 | 6629912 | 6024 | 67.18 | C | registered |
|  | B_vietnamiensis_G4_uid58075 | 3 | 5 | 8391070 | 7606 | 65.74 | C | registered |
|  | B_dolosa_AUO158_uid54351 | ND | ND | 6420486 | 4822 | 66.81 | UC | registered |
|  | B_pyrrocinia_CH_67_uid199595 | ND | ND | 8055291 | 7230 | 67.34 | UC | local annotation |
|  | B_ubonensis_Bu_uid54793 | ND | ND | 6981638 | 7192 | 67.03 | UC | registered |
|  | B_TJI49_uid179699 | ND | ND | 7512372 | 8940 | 66.26 | UC | registered |
| mallei | B_pseudomallei_1026a_uid180043 | ND | ND | 7169326 | 6148 | 68.09 | UC | registered |
|  | B_pseudomallei_1026b_uid162511 | 2 | 0 | 7231415 | 6070 | 68.14 | C | registered |
|  | B_pseudomallei_1106a_uid58515 | 2 | 0 | 7089249 | 7174 | 68.26 | C | registered |
|  | B_pseudomallei_1106b_uid54361 | ND | ND | 7220103 | 7192 | 68.19 | UC | registered |
|  | B_pseudomallei_112_uid54769 | ND | ND | 6973774 | 8050 | 67.75 | UC | registered |
|  | B_pseudomallei_1258a_uid180044 | ND | ND | 6778455 | 5816 | 68.06 | UC | registered |
|  | B_pseudomallei_1258b_uid180045 | ND | ND | 7091219 | 6060 | 68.09 | UC | registered |
|  | B_pseudomallei_14_uid54781 | ND | ND | 6788724 | 8255 | 67.37 | UC | registered |
|  | B_pseudomallei_1655_uid54283 | ND | ND | 7044524 | 6294 | 67.99 | UC | registered |
|  | B_pseudomallei_1710a_uid54285 | ND | ND | 7337754 | 7497 | 67.99 | UC | registered |
|  | B_pseudomallei_1710b_uid58391 | 2 | 0 | 7308054 | 6344 | 67.98 | C | registered |
|  | B_pseudomallei_305_uid54601 | ND | ND | 7455154 | 7371 | 67.85 | UC | registered |
|  | B_pseudomallei_354a_uid180009 | ND | ND | 7216777 | 6506 | 67.93 | UC | registered |
|  | B_pseudomallei_354e_uid180046 | ND | ND | 7125878 | 6159 | 68.1 | UC | registered |
|  | B_pseudomallei_406e_uid54371 | ND | ND | 7421446 | 6363 | 68.05 | UC | registered |
|  | B_pseudomallei_576_uid55425 | ND | ND | 7247596 | 7395 | 68.08 | UC | registered |
|  | B_pseudomallei_668_uid58389 | 2 | 0 | 7040403 | 7116 | 68.29 | C | registered |
|  | B_pseudomallei_7894_uid54771 | ND | ND | 7045674 | 8315 | 67.3 | UC | registered |
|  | B_pseudomallei_91_uid54779 | ND | ND | 6940414 | 8331 | 67.38 | UC | registered |
|  | B_pseudomallei_9_uid54777 | ND | ND | 6881670 | 8224 | 67.42 | UC | registered |
|  | B_pseudomallei_B7210_uid54773 | ND | ND | 6952882 | 8122 | 67.56 | UC | registered |
|  | B_pseudomallei_BCC215_uid54765 | ND | ND | 7044657 | 7939 | 67.75 | UC | registered |
|  | B_pseudomallei_BPC006_uid174460 | 2 | 0 | 7155061 | 7158 | 68.2 | C | registered |
|  | B_pseudomallei_DM98_uid54783 | ND | ND | 6790566 | 8581 | 67.01 | UC | registered |
|  | B_pseudomallei_K96243_uid57733 | 2 | 0 | 7247547 | 5727 | 68.06 | C | registered |
|  | B_pseudomallei_MARAN_uid199962 | ND | ND | 6675393 | 7302 | 67.39 | UC | local annotation |
|  | B_pseudomallei_MSHR1043_uid195201 | ND | ND | 7223030 | 6297 | 68.11 | UC | registered |
|  | B_pseudomallei_NCTC_13177_uid54767 | ND | ND | 7170038 | 8109 | 67.76 | UC | registered |
|  | B_pseudomallei_NCTC_13392_uid199947 | ND | ND | 7166836 | 7542 | 68.14 | UC | local annotation |
|  | B_pseudomallei_Pakistan_9_uid55843 | ND | ND | 7150715 | 7491 | 68.1 | UC | registered |
|  | B_pseudomallei_Pasteur_52237_uid54289 | ND | ND | 7364297 | 6629 | 68.01 | UC | registered |
|  | B_pseudomallei_S13_uid54287 | ND | ND | 7401609 | 6761 | 68.08 | UC | registered |
|  | B_mallei_2002721280_uid54379 | ND | ND | 5703758 | 4847 | 68.32 | UC | registered |
|  | B_mallei_ATCC_10399_uid54279 | ND | ND | 5940749 | 5276 | 68.44 | UC | registered |
|  | B_mallei_ATCC_23344_uid57725 | 2 | 0 | 5835527 | 5023 | 68.49 | C | registered |
|  | B_mallei_FMH_uid54291 | ND | ND | 5899820 | 5169 | 68.47 | UC | registered |
|  | B_mallei_GB8_horse_4_uid54281 | ND | ND | 5812598 | 5888 | 68.42 | UC | registered |
|  | B_mallei_JHU_uid54293 | ND | ND | 5894380 | 5128 | 68.41 | UC | registered |
|  | B_mallei_NCTC_10229_uid58383 | 2 | 0 | 5742303 | 5509 | 68.47 | C | registered |
|  | B_mallei_NCTC_10247_uid58385 | 2 | 0 | 5848380 | 5415 | 68.48 | C | registered |
|  | B_mallei_PRL_20_uid54653 | ND | ND | 5681490 | 5374 | 68.31 | UC | registered |
|  | B_mallei_SAVP1_uid58387 | 2 | 0 | 5232401 | 5184 | 68.4 | C | registered |
|  | B_thailandensis_Bt4_uid54787 | ND | ND | 6590481 | 6771 | 67.46 | UC | registered |
|  | B_thailandensis_E264_uid54655 | ND | ND | 6354383 | 5651 | 67.78 | UC | registered |
|  | B_thailandensis_E264_uid58081 | 2 | 0 | 6723972 | 5632 | 67.63 | C | registered |
|  | B_thailandensis_MSMB121_uid201037 | 2 | 0 | 6731379 | 5758 | 67.51 | C | registered |
|  | B_thailandensis_MSMB43_uid182042 | ND | ND | 7248569 | 6198 | 67.18 | UC | registered |
|  | B_thailandensis_MSMB43_uid54775 | ND | ND | 7009013 | 7426 | 66.75 | UC | registered |
|  | B_thailandensis_TXDOH_uid54795 | ND | ND | 6550698 | 6720 | 67.48 | UC | registered |
|  | B_oklahomensis_C6786_uid54789 | ND | ND | 7014783 | 6979 | 66.92 | UC | registered |
|  | B_oklahomensis_EO147_uid54791 | ND | ND | 7085212 | 7235 | 66.72 | UC | registered |
| out | B_RPE64_uid205541 | 3 | 2 | 6964487 | 6498 | 63.15 | C | registered |
|  | B_YI23_uid81081 | 3 | 3 | 8896411 | 7804 | 63.26 | C | registered |
|  | B_SJ98_uid160003 | ND | ND | 7879286 | 7268 | 62.68 | UC | registered |
|  | B_CCGE1002_uid42523 | 3 | 1 | 7884858 | 6888 | 63.27 | C | registered |
|  | B_JPY251_uid199221 | ND | ND | 8614723 | 7888 | 63.03 | UC | JGI |
|  | B_H160_uid55101 | ND | ND | 7906641 | 7460 | 62.82 | UC | registered |
|  | B_WSM4176_uid199219 | ND | ND | 9065763 | 8366 | 62.93 | UC | JGI |
|  | B_kururiensis_M130_uid199910 | ND | ND | 7129201 | 6100 | 65.02 | UC | local annotation |
|  | B_BT03_uid180532 | ND | ND | 10671621 | 10126 | 61.85 | UC | registered |
|  | B_terrae_BS001_uid168186 | ND | ND | 11308219 | 10234 | 61.78 | UC | registered |
|  | B_phymatum_STM815_uid58699 | 2 | 2 | 8676562 | 7495 | 62.29 | C | registered |
|  | B_phytofirmans_PsJN_uid58729 | 2 | 1 | 8214658 | 7236 | 62.29 | C | registered |
|  | B_xenovorans_LB400_uid57823 | 3 | 0 | 9731138 | 8702 | 62.63 | C | registered |
|  | B_Ch1_1_uid48975 | ND | ND | 8743657 | 7743 | 62.44 | UC | registered |
|  | B_CCGE1003_uid46253 | 2 | 0 | 7043595 | 5988 | 63.25 | C | registered |
|  | B_CCGE1001_uid42975 | 2 | 0 | 6833751 | 5960 | 63.63 | C | registered |
|  | B_phenoliruptrix_BR3459a_uid176370 | 2 | 1 | 7651131 | 6496 | 63.15 | C | registered |
|  | B_graminis_C4D1M_uid54887 | ND | ND | 7480230 | 6747 | 62.86 | UC | registered |

^a^ Indicates “completed”.

^b^ Indicates “not determinant”.

^c^ Indicates “uncompleted”.

**Table S2. Genes involved in Type III secretion among *Burkholderia glumae* BGR1, *B. gladioli* BSR3, and *B. plantarii* ATCC 43733^T^.**

| Type III main cluster | B. *glumae* BGR1 | *B. gladioli BSR3* | *B. plantarii* ATCC 43733^T^ | *B. glumae* PG1 |
| --- | --- | --- | --- | --- |
| YscC; type III secretion protein SctC | bglu_2g02480 | bgla_2g02870 | bpln_2g02740 | AJK48327.1 |
| YscJ; type III secretion protein SctJ | bglu_2g02410 | bgla_2g02800 | bpln_2g02670 | AJK48320.1 |
| YscL; type III secretion protein SctL | bglu_2g02430 | bgla_2g02820 | bpln_2g02690 | AJK48322.1 |
| YscN; ATP synthase in type III secretion protein S | bglu_2g02440 | bgla_2g02830 | bpln_2g02700 | AJK48323.1 |
| YscQ; type III secretion protein SctQ | bglu_2g02350 | bgla_2g02740 | bpln_2g02610 | AJK48314.1 |
| YscR; type III secretion protein SctR | bglu_2g02340 | bgla_2g02730 | bpln_2g02600 | AJK48313.1 |
| YscS; type III secretion protein SctS | bglu_2g02330 | bgla_2g02720 | bpln_2g02590 | AJK48312.1 |
| YscT; type III secretion protein SctT | bglu_2g02460 | bgla_2g02850 | bpln_2g02720 | AJK48325.1 |
| YscU; type III secretion protein SctU | bglu_2g02380 | bgla_2g02770 | bpln_2g02640 | AJK48317.1 |
| YscV; type III secretion protein SctV | bglu_2g02370 | bgla_2g02760 | bpln_2g02630 | AJK48316.1 |
| putative awr type III effector family protein | bglu_2g02250 | bgla_2g02070 | bpln_2g02510 | AJK48304.1 |
| Transcriptional regulator, winged helix family | bglu_2g02260 | bgla_2g02080 | bpln_2g02520 | AJK48305.1 |
| HpaB | bglu_2g02270 | bgla_2g02090 | bpln_2g02530 | AJK48306.1 |
| Hypothetical protein | bglu_2g02280 | bgla_2g02100 | bpln_2g02540 | AJK48307.1 |
| type III secretion protein SctE | bglu_2g02290 | bgla_2g02110 | bpln_2g02550 | AJK48308.1 |
| type III secretion protein SctD | bglu_2g02300 | bgla_2g02120 | bpln_2g02560 | AJK48309.1 |
| type III effector protein HrpW | bglu_2g02490 | bgla_2g02880 (partial match) | bpln_2g02750 | AJK48328.1 |
| type III secretion protein HpaP | bglu_2g02360 | bgla_2g02750 | bpln_2g02620 | AJK48315.1 |
| type III secretion protein HrpB1/HrpK | bglu_2g02390 | bgla_2g02780 | bpln_2g02650 | AJK48318.1 |
| type III secretion protein HrpB2 | bglu_2g02400 | bgla_2g02790 | bpln_2g02660 | AJK48319.1 |
| type III secretion protein HrpB4 | bglu_2g02420 | bgla_2g02810 | bpln_2g02680 | AJK48321.1 |
| type III secretion protein HrpB7 | bglu_2g02450 | bgla_2g02840 | bpln_2g02710 | AJK48324.1 |
| type III secretion system response regulator HrpG | bglu_2g02500 | bgla_2g02890 | bpln_2g02760 | AJK48329.1 |
| type III effector HrpK | bglu_2g02530 | bgla_2g02920 | bpln_2g02790 | AJK48332.1 |
| HrpB | bglu_2g02470 | bgla_2g02860 | bpln_2g02730 | AJK48326.1 |

**Table S4. Genes involved in tropolone biosynthesis in *B. plantarii* ATCC 43733^T^.**

| Locus ID | B.glumae PG1 | Annotation |
| --- | --- | --- |
| bpln_1g07710 | AJK45324.1 | LysR family transcriptional regulator |
| bpln_1g07720 | AJK45325.1 | AHL synthase |
| bpln_1g07790 | AJK45332.1 | LuxR family transcriptional regulator |
| bpln_1g07810 | AJK45334.1 | Thioesterase superfamily protein |
| bpln_1g07820 | AJK45335.1 | Acyl-CoA dehydrogenase |
| bpln_1g07830 | AJK45336.1 | Chorismate mutase |
| bpln_1g29230 | AJK47497.1 | Phosphopantothenoylcysteine synthase/decarboxylase |
| bpln_2g15430 | AJK49535.1 | Glutathione S-transferase domain protein |

**Table S5. Genes involved in rhizotoxin biosynthesis among bacteria strains.**

| *Xanthomonas oryzae* pv. *oryzae* KACC10331 | *B. plantarii* ATCC 43733^T^ | *B.glumae* PG1 | *B. phenoliruptrix* | *B. phymatum* | *B. JYP251* |
| --- | --- | --- | --- | --- | --- |
| YP_200026 | bpln_2g20640 | AJK50103.1 | YP_006836513 | YP_001863717 | bj251_4558lm |
| (Glutamine synthetase) | 71.5% (321/449)^a^ | 71.5% (321/449) | 68.0% (306/450) | 67.8% (305/450) | 67.3% (303/450) |
| YP_200027 | bpln_2g20630 | AJK50102.1 | YP_006836512 | YP_001863716 | bj251_4557lm |
| (Homoserine O-succinyltransferase) | 64.0% (187/292) | 64.0% (187/292) | 59.5% (175/294) | 58.5% (172/294) | 57.2% (167/292) |
| YP_200028 | bpln_2g20590 | AJK50098.1 | YP_006836509 | YP_001863713 | bj251_4554lm |
| (RtxC) | 71.3% (243/341) | 71.0% (242/341) | 68.3% (233/341) | 68.3% (233/341) | 69.2% (236/341) |
| YP_200029 | bpln_2g20580 | AJK50097.1 | YP_006836508 | YP_001863712 | bj251_4553l |
| (RtxA) | 79.5% (350/440) | 80.0%(351/440) | 75.9% (325/428) | 74.1% (326/440) | 74.8% (329/440) |
| YP_200030 | bpln_2g20570 | AJK50096.1 | YP_006836507 | YP_001863711 | bj251_4552lm |
| (Hypothetical protein) | 65.6% (252/384) | 65.6% (252/384) | 63.4% (232/366) | 62.8% (230/366) | 61.6% (223/362) |

^a^ Represents identities of amino acid sequences.
