# Supplementary figures and images for: Comparative genome analysis of rice-pathogenic Burkholderia provides insight into capacity to adapt to different environments and hosts
Source: BMC Genomics. 2015 May 6;16(1):349. doi: 10.1186/s12864-015-1558-5 (PMC4422320; doi:10.1186/s12864-015-1558-5)

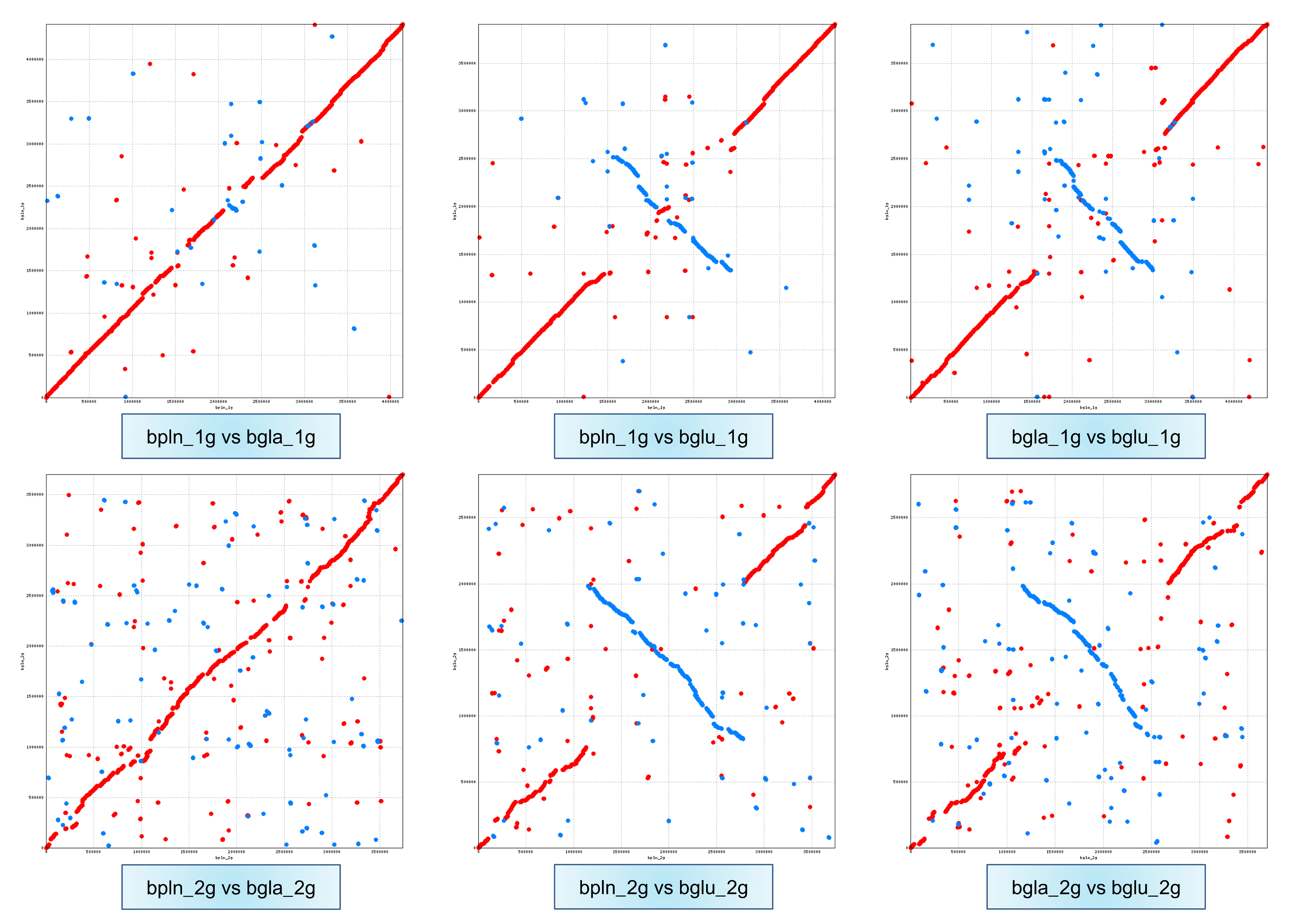

Supplement: Additional file 3: Figure S1. — MUMmer analysis of each chromosome between Burkholderia glumae BGR1, B. gladioli BSR3, and B. plantarii ATCC 43733T. [file 12864_2015_1558_MOESM3_ESM.png]

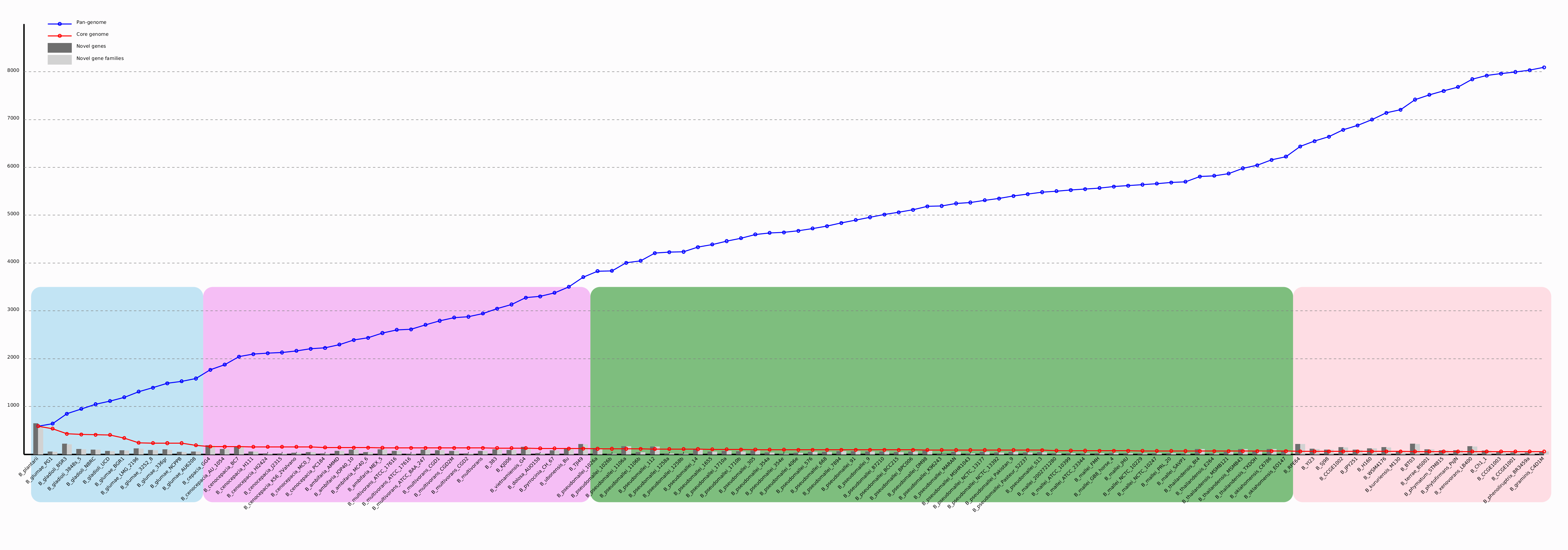

Supplement: Additional file 4: Figure S2. — Pan-genome and core-genome analysis based on 106 genomes of Burkholderia strains (listed in Additional file 1: Table S1). The blue box, violet box, green box, and pink box represent the glumae group, cepacia group, mallei group, and outgroup, respectively. Each group is designated in Additional file 1: Table S1 and Additional file 5: Figure S3. [file 12864_2015_1558_MOESM4_ESM.png]

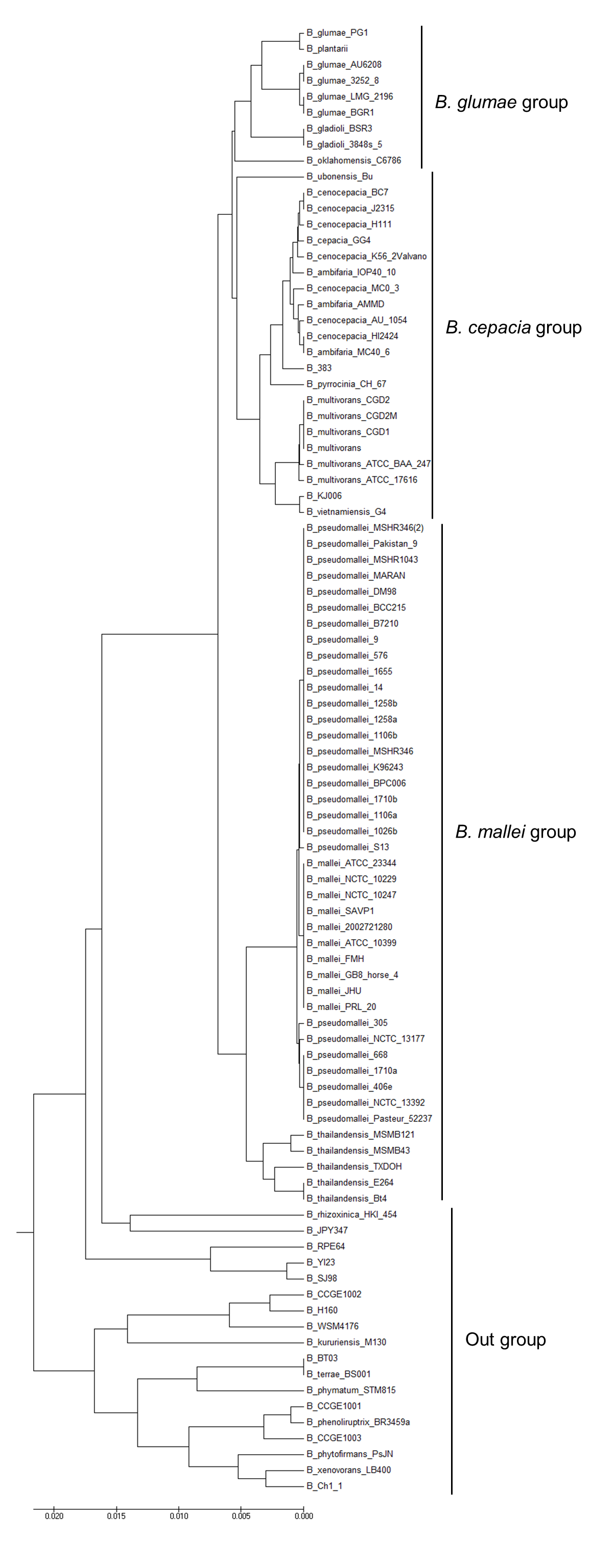

Supplement: Additional file 5: Figure S3. — Phylogenetic tree of 106 Burkholderia species based on 16S rRNA sequences. [file 12864_2015_1558_MOESM5_ESM.png]
